# Gender Was Associated with Depression but Not with Gastrointestinal Dysfunction in Patients with Parkinson's Disease

**DOI:** 10.1155/2021/3118948

**Published:** 2021-12-22

**Authors:** Qin Xiao-ling, Du Yin-zhen, Liu Xue-kui, Li Xue, Cheng Gang, Li Zai-li, Gao Dian-shuai

**Affiliations:** ^1^Department of Geriatrics, Shanghai Fourth People's Hospital, Tongji University School of Medicine, Shanghai 200434, China; ^2^Xuzhou Key Laboratory of Neurobiology, Department of Neurobiology and Anatomy, Xuzhou Medical University, Xuzhou 221004, Jiangsu, China; ^3^Xuzhou Clinical School of Xuzhou Medical University, No. 199 Jiefang Road, Xuzhou 221009, Jiangsu, China

## Abstract

**Objective:**

To investigate the association between gender and gastrointestinal (GI) dysfunctions, as well as gender and other motor symptoms/nonmotor symptoms, in a sample of PD patients.

**Methods:**

186 patients with PD were recruited into this study and divided into male PD group (M-PD) and female PD group (FM-PD). Demographic and PD-related clinical information of the participants were collected by the same neurologist. PD patients were objectively assessed by a spectrum of rating scales of motor symptoms and nonmotor symptoms (including GI dysfunctions). The data were analyzed by SPSS 20 statistical software.

**Results:**

Totally 95 cases (51.08%) were in the M-PD group and 91 cases (48.92%) in the FM-PD group. There were no significant differences in age, BMI, and lifestyles between the two groups (*P* > 0.05). Males had higher educational level (*P* = 0.002). Females were more likely to have early satiety and loss of appetite (*P* = 0.025, *P* = 0.001). There were no significant differences in LED disease duration, age of motor symptoms onset, types of motor symptoms onset, location of motor symptoms onset, and phenotype of motor symptoms between the two groups (*P* > 0.05). Females had significantly higher UPDRS-III and HAMD scores than males (*P* = 0.037, *P* = 0.034). There were no significant differences in PQSI, ESS, RLS, RBD, HAMA, HAMD, and MoCA scores between the two groups. Gender was associated with HAMD (OR = 0.682, *P* = 0.019).

**Conclusions:**

Gender is a risk factor for depression, but not for GI dysfunctions in patients with PD.

## 1. Introduction

Parkinson's disease (PD) is the second most common, age-related neurodegenerative disorder. The classical pathological feature of PD is the progressive degeneration of the nigrostriatal DA pathway, which leads to the primary motor symptoms, including resting tremor, bradykinesia, rigidity, and gait disturbances [1]. Nonmotor symptoms of the disorder, equally common, important, and distressing, include autonomic dysfunctions, cognitive abnormalities, psychiatric symptoms, and sleep disorders, among which gastrointestinal (GI) dysfunctions are the most common and greatly decrease the life quality of the patients and increase overall disability. The spectrum of PD-associated GI dysfunction includes oral problems, salivation or drooling, dysphagia, gastroesophageal reflux, gastroparesis (nausea, vomiting, early satiety, and loss of appetite), and bowel dysfunction (constipation, and, occasionally, loose stool and fecal incontinence, among others) [2]. GI dysfunctions may already be present before motor symptoms and approximately 60–80% of patients with PD eventually experience GI symptoms [3]. The topic of PD-related GI dysfunctions has attracted more and more attention because it contributes to a deeper understanding of the development of PD.

Pathological abnormalities of the disease including the PD-related aggregation of alpha synuclein (*α*SYN) have been identified in intestinal biopsies from PD patients, and the deposition of *α*SYN appeared within the enteric nervous system (ENS) before it was observed in the central nervous system (CNS) [4]. The gut microbiota is required for *α*SYN pathology and is a hallmark of motor and GI dysfunction in PD model [5]. PD patients exhibit significant differences in gut bacterial populations and inflammation and immune activities [5]. It has been proposed that PD pathology may originate in the gut, from ingestion of an external pathogen that provokes local inflammation, producing leakiness in the mucosal barrier, which then permits entry and ongoing damage in the gut with deposition of *α*SYN in the ENS, and later spread to the CNS [5].

Biological gender is increasingly recognised and widely discussed as one of the important factors (aging, genetics, environment, and immune status) which influence the development of PD. There are clear gender-related differences in epidemiological and clinical features of the disease. Studies have revealed the male as a prominent risk factor for developing PD and male to female ratios for incidence rates vary from 1.37 to 3.7 [6]. Moreover, males and females are different in mortality rate, disease progression, clinical profile (motor and nonmotor symptoms), response to pharmacological therapies and deep brain stimulation procedure (DBS) [7]. Although the present research results are not completely consistent, it is an indisputable fact that gender differences exist in PD.

The etiology behind gender differences may be multiple factors, including the neuroprotective effect of estrogen, genetic factors, differences in brain organisation, structure and function, and differences in environmental exposures and lifestyle factors [8]. As mentioned above, inflammation and immunity in gut play a pivotal role in the development of PD. Researchers found a disease-associated increase in numerous immune mediators in the stool of female PD patients, while the stool of male patients did not differ significantly from controls [9]. Intestinal inflammation is consistent with intestinal symptoms and may be a driver of disease pathology [9].

On the basis of the above, we were very interested in exploring and understanding the possible association between gastrointestinal symptoms and gender in patients with PD. The study was therefore designed.

## 2. Methods

### 2.1. Patients

A total of 186 idiopathic PD patients were recruited from the clinic or Impatient Department of Neurology, Xuzhou Central Hospital/Clinical Hospital of Xuzhou Medical University from March 2017 to December 2019. The clinical diagnosis of idiopathic PD was determined based on the MDS clinical diagnostic criteria for Parkinson's disease [10]. Subjects with other diseases, such as respiratory diseases, urinary system diseases, cardiovascular and cerebrovascular diseases, and primary mental disorders, were excluded (but patients with cognitive disorder are not deliberately excluded). Subjects who were unable to finish the questionnaire were also excluded.

The protocol was approved by the Ethics Committee of Xuzhou Clinical Hospital of Xuzhou Medical University. All participants completed the written informed consents.

### 2.2. Clinical Assessments

#### 2.2.1. Demographic and General Information

A movement disorder specialist clinically evaluated PD subjects in an “ON” state. Demographic variables, including gender, age, educational level, height, weight, body mass index (BMI), and lifestyle (occupational exposure to insecticidal/herbicides, smoking, and alcohol drinking), were recorded for all participants. General clinical information, including levodopa-equivalent daily dose (LEDD, calculated based on previously reported conversion factors [11]), and disease duration, was also recored.

### 2.3. Assessment of GI Dysfunction

GI dysfunction is a common group of nonmotor symptoms in PD. In this study, we mainly focused on mouth pain, dry mouth, dysgeusia, dysphagia, drooling, gastroparesis (including early satiety, loss of appetite, nausea, and vomiting), and bowel dysfunction (including constipation and, occasionally, loose stool and fecal incontinence).

The Scale for Outcomes in Parkinson's Disease-Autonomic (SCOPA-AUT) [12] and Parkinson's Disease Nonmotor Symptoms Scale (PD-NMSS) [13] were carried out in all patients. Dysphagia/choking (item 1 of SCOPA-AUT), drooling (item 2 of SCOPA-AUT), early satiety (item 4 of SCOPA-AUT), constipation (item 5 of SCOPA-AUT), loose stools (item 6 of SCOPA-AUT), fecal incontinence (item 7 of SCOPA-AUT), and dysgeusia (item 28 of SCOPA-AUT) were recorded as “Yes” (over grade (2) or “No” (never)). Other symptoms, including loss of appetite, dry mouth, mouth pain, nausea, and vomiting, were surveyed according to PD-NMSS.

### 2.4. Assessment of Motor Symptoms

Age of motor symptom onset and disease duration (equivalent to duration of motor signs) were recorded for all participants. The severity of PD was assessed by Hoehn-Yahr (H&Y) stage and the Unified Parkinson's Disease Rating Scale (UPDRS). Motor symptoms of PD patients were evaluated by UPDRS III [14]. Types of motor symptoms onset were divided into tremor and nontremor (including bradykinesia, rigidity, and gait disturbances). Onset locations were marked as Left (left upper or lower limbs), Right (right upper or lower limb), and others (bilateral or head). Motor symptom phenotypes were identified based on the ratio of the mean tremor score (sum of items 20 and 21 in the UPDRS III divided by four) to the mean bradykinesia/rigid score (sum of items 22–27 and 31 in the UPDRS III divided by 15). Patients with a ratio greater than 1.0, less than 0.80, and between 0.80 and 1.0 were classified into the tremor-dominant (TD) subtype, akinetic-rigid-dominant (AR) subtype, and mixed subtype, respectively [15].

### 2.5. Assessment of Other Nonmotor Symptoms

Each participant also completed the Pittsburgh Sleep Quality Index (PSQI), Epworth Sleepiness Scale (ESS), Rapid Eye Movement Sleep Behavior Disorder Screening Questionnaire (RBD-SQ), restless leg syndrome (RLS) diagnosis, Hamilton's Depression Scale (HAMD), Hamilton's Anxiety Scale (HAMA), and Montreal Cognitive Assessment (MoCA).

The PSQI is a self-report questionnaire that assesses nighttime sleep over 1 month, with a global score ranging from 0 to 21. Higher scores represent poorer subjective sleep quality [16]. The ESS is a widely used questionnaire for assessing the general level of daytime sleepiness. The ESS is composed of eight items that address typical day-to-day situations. Each item ranges from 0 to 3 points (0 = would never doze, 3 = high chance of dozing) to yield a total ESS score of 0–24 (lowest to highest sleep propensity). Subjects with ESS > 10 were considered excessive daytime sleepers (EDS), and normal sleep propensity was 0–10 [17].

The RBD-SQ is a valuable tool for screening Rapid Eye Movement (REM) Sleep Behavior Disorder (RBD) [18]. A RBD score of 5 or greater was defined as probable RBD. Diagnosis of RLS was made according to the RLS diagnostic criteria proposed by the International Restless Legs Syndrome Study Group (IRLSSG) in 2014, which is based on four essential features of the questionnaire after the exclusion of RLS mimics, such as positional discomfort, muscle cramp, venous stasis, vascular claudication, and peripheral neuropathy [19].

HAMD and HAMA are frequently used to quantify severity of depression and anxiety, respectively [20]. MoCA is the most widely used by frontline physicians to clinically assess cognitive functions. All scales used in this study have been validated in Chinese.

### 2.6. Statistical Analysis

The measurement data are expressed as mean ± SD (standard deviation), and the enumeration data are shown as numbers (rate). Two independent sample *t*-tests were used to analyze the measurement data of two groups with a normal distribution. Nonnormal distribution data were analyzed using nonparametric tests (Mann-Whitney test). The enumeration data were analyzed using the *χ*^2^ test. Logistic regression analysis was used to assess the correlations between gender and GI dysfunctions and other motor/nonmotor symptoms in PD patients. *P* < 0.05 was considered statistically significant. Statistical analysis was conducted mainly between the M-PD and FM-PD group in this study.

## 3. Results

### 3.1. Demographic Data of PD Patients

A total of 186 idiopathic PD patients were ultimately included in this study (T-PD, total PD group). The mean age was 67.53 ± 8.52 years old (range: 42–87 years) with a mean disease duration of 68.11 ± 69.59 months (range: 3–420 months). Of the total PD patients, 95 male patients (51.08%) were in the male PD (M-PD) group and 91 female patients (48.92%) in the female PD (FM-PD) group. The general information of these three groups was listed in Table 1.

Male patients had higher educational level than Female patients (*P* = 0.002). There were no significant differences in age, BMI, lifestyle, and LEDD between the two groups.

### 3.2. GI Dysfunctions

In T-PD, the most common GI dysfunctions were “dry mouth” and “constipation,” 59.14% and 54.84%, respectively. The same characteristics were found in both FM-PD and M-PD groups. 55 (57.89%) patients in M-PD and 55 (60.44%) in FM-PD had “dry mouth,” but there was no significant difference between the two groups (*χ*^2^ = 0.1246, *P* = 0.724). “Constipation” in FM-PD group (54, 59.34%) was higher than that in M-PD (48, 50.52%), but no statistically significant difference existed between the two groups (*χ*^2^ = 0.1694, *P* = 0.681).

Two of the GI dysfunctions were significantly different between M-PD and FM-PD. 28 (30.8%) female patients had “early satiety,” significantly higher than that of male patients (*χ*^2^ = 4.99, *P* = 0.025). 42 (46.15%) female patients had “loss of appetite,” also significantly higher than that of male patients (22, 23.16%) (*χ*^2^ = 18.89, *P* = 0.001). “Early satiety” and “loss of appetite” are both common symptoms of gastroparesis. It was concluded that female PD patients were more likely to present with gastroparesis symptoms. There were no significant differences between M-PD and FM-PD in “mouth pain,” “dysgeusia,” “dysosmia,” “dysphagia,” “nausea,” “drooling,” “vomiting,” “loose stool,” and “fecal incontinence.” (Table 2).

### 3.3. Motor Symptoms and Other Nonmotor Symptoms

Motor and other nonmotor symptoms of T-PD, M-PD, and FM-PD groups are listed in Table 3.

In terms of motor symptom features, there were no differences in age of motor symptoms onset, disease duration, onset type, onset location, phenotype of motor symptoms, and H-Y stage between male and female patients. However, female patients had significantly higher UPDRS III score than male patients. (*χ*^2^ = −2.131, *P* = 0.034).

In terms of other nonmotor symptoms, there were no significant differences in sleep disorders (including PQSI score, ESS scores, and RBD), in cognitive function (MoCA evaluation), and in HAMA scores between male and female patients. But the scores of HAMD were significantly higher in female patients than male patients (*χ*^2^ = −2.373, *P* = 0.033).

### 3.4. Symptoms Associated with Gender

Logistic regression analysis was performed on all variables (Table 4). We assigned values by gender (female = 0, male = 1). Ultimately, the HAMD score was found to decrease with each one-unit increase in gender (from 0 to 1) (OR = 0.682; *P* = 0.019). As a result, gender is a risk factor for depression, but not for GI dysfunctions in patients with PD.

## 4. Discussion

Our research focused on the relationship between gender and GI dysfunctions and other motor/nonmotor symptoms in PD patients in the Chinese population. It showed that female patients were more likely to be less educated. Dry mouth and constipation were the most common GI dysfunctions in Chinese PD patients, while gastroparesis was significantly more common in female patients than male patients. Female patients had more severe motor dysfunction and were more likely to suffer from depression. In addition, although gender was ultimately proven to be a risk factor for depression, there was no correlation between gender and GI dysfunctions (including gastroparesis), which was inconsistent with our hypothesis.

As an overlooked autonomic symptom of Parkinson's disease, dry mouth has rarely been systematically described, despite of an involvement of salivary glands by *α*SYN pathology in the disease. One study reported that 60.8% of PD patients had dry mouth, and only 27.9% of controls (*p* < 0.0001), which might be an early manifestation of autonomic involvement in PD. The study also pointed out that dry mouth and drooling coexisted in 30% of the cases [21]. However, in another study, 50% of the PD patients were reported to have xerostomia accompanied by hyposalivation [22]. It was reported by other researchers that an even higher prevalence of 69% PD patients had sicca symptoms (xerostomia and dry eyes), which might be caused by anti-PD medications or antidepressant drugs [23]. Our study showed that 59.14% of the total PD patients had dry mouth, 57.89% in M-PD and 60.44% in FM-PD, respectively, in accordance with other reports. However, there was no significant difference between men and women. Dry mouth contributes to both caries and periodontal disease, influences not only oral health but also general health-related quality of life (QoL), and is thought to be closely associated with malnutrition in PD patients [23].

Constipation, generally recognised as one of the most common GI dysfunctions, and as one of the risk factors of PD, with its incidence ranging from 50 to 80%, has attracted more and more extensive attention from researchers in recent years [24]. Several studies have demonstrated that constipation may precede the occurrence of motor symptoms underlying an earlier involvement of the ENS and the dorsal motor nucleus of the vagus in the *α*SYN pathology. It is mainly due to slower colonic transit or puborectalis dyssynergia, but the concomitant use of antiparkinsonian, pain, and antidepressant medications may worsen it [25].

Our study confirmed that constipation was one of the most common GI dysfunctions in both female and male patients. Although female patients had a significantly higher incidence than male patients, no statistical difference was found between female and male patients. The conclusion was inconsistent with previous research [24], possibly due to the different sample sizes. The study showed that female PD patients presented with more “early satiety” and “loss of appetite,” which are among the most common symptoms of gastroparesis [26]. Gastroparesis is rare with a prevalence of approximately 0.2% in the general population; the common causes of gastroparesis include diabetes, surgery, and idiopathy [27]. In a report on 146 patients with gastroparesis, idiopathic gatroparesis is the most common, while PD was the fourth leading cause of gastroparesis [28]. Since females in general have slower gastric emptying than males, gastroparesis occurs more often in female patients than in male patients by a 3 : 1 margin [29]. Few formal prevalence studies of gastroparesis have been performed in PD patients, but reports involving small samples of patients have provided frequency estimates, ranging from 35 to 100% [30]. In our study, more than one-third of PD patients had symptoms of gastroparesis, and the ratio of female to male patients with PD gastroparesis was nearly 2 : 1. It fully suggested that the incidence of gastroparesis in PD patients is high, and female patients were more likely to suffer from gastroparesis.

Diagnosis of gastroparesis formally depends on the combination of symptoms and delayed gastric emptying (DGE), which is chiefly characterized by reduced stomach motility in the absence of mechanical obstruction [27]. The mechanism of gastroparesis is not yet to be fully elucidated. It may mainly involve autonomic and intrinsic neuropathies of excitatory and inhibitory systems in the gut, including damage to the vagal nerve and impairment of the interstitial cells of Cajal, which regulate smooth muscle cell contractility [31]. Normal digestion in the stomach and gastric emptying are mediated by multiple hormones, including gastrin, leptin, motilin, and ghrelin. Addtionally, the vagal nerve and the ENS play key roles in mediating gastric emptying [32]. In patients with PD, oral levodopa treatment, as well as anticholinergic medications, may also contribute to gastroparesis in a dose-dependent manner, which in turn impedes levodopa gastrointestinal absorption, hence lowering its plasma concentration and contributing to motor fluctuations, and, when impeding food nutrients absorption, resulting in malnutrition. Treatment that circumvent the GI tract (such as apomorphine injection, levodopa intestinal gel delivery, levodopa inhalation powder, and deep brain stimulation.) and prokinetic agents (5-HT agonist) might impair oral levodopa absorption and efficacy in PD patients [33].

Motor symptoms are fundamental to the diagnosis of PD. Over the past decades, the effect of biological gender on the presentation of PD motor symptoms (phenotype and severity) has been broadly discussed. Female patients were reported to present more often with tremor [34], later emerging of motor symptoms and higher propensity to develop postural instability [35]. Male patients, on the other hand, had been recently associated with later development of freezing gait [7]. In our study, female patients were reported to have more severity of motor symptoms than male patients. However, there were no differences in age of motor symptoms onset, onset symptom, onset location, and phenotype. The result was not entirely consistent with some previous studies, but was consistent with Danielle S. Abraham's, which reported that females had more advanced Hoehn and Yahr stage and worse disability [7]. The underlying reasons for the different results need further investigations.

Females presented more depression than males among a host of other nonmotor symptoms, including sleep disorders and cognitive impairment. Depression affects up to 50% of patients with PD but is overlooked in over 60% of patients and often undertreated [36]. In a study of 951 PD patients, researchers similarly concluded depression was more common in female patients. However, other nonmotor symptoms such as fatigue and restless legs were also reported to be more common in females [37]. Given that depression is often missed by neurologists, our study emphasizes the need to screen PD patients, especially females, for depression.

We spotlighted in this study that gender was associated with depression, but not with GI dysfunctions in PD patients. In general populations, females are as well at significantly greater risk to have depression than males [38]. Researchers pointed out that sex alters brain function by hormonal effects, genomic sex effects, and environment effects, and the brain itself has sex difference in structure, cell activity/signaling, and network [39]. As for pathophysiology of depression in PD, the mesolimbic dopaminergic pathway and the complex network of interrelated systems, such as the serotonergic system, have been suggested to contribute to the development of depression [40]. Serotonergic system is altered particularly in limbic areas (including the temporal cortex and hippocampus), frontal regions, and the raphe nuclei [41]. Serotonin is a hormone and an excitatory neurotransmitter that is produced in large quantities in the GI tract but is most prominently known for its central contributions to anxiety and depression. Clarke and colleagues pioneered to report in 2013 that the early life gut microbiome regulates the hippocampal serotonergic system in a sex-dependent manner, and may be in a humoral route. In their study, male germ-free (GF) animals have a significant elevation in the hippocampal concentration of 5-hydroxytryptamine (5-HT) and 5-hydroxyindoleacetic acid (5-HIAA), and in plasma level of tryptophan, compared to controls. These findings opened a door to shed light on the links between gut microbiota and brain neurotransmitters [42]. This consideration aligned with the evidence that regulation of the brain serotonergic system is sexually dimorphic and contributed to growing efforts to examine sex as a biological variable [43]. Our study suggested that female PD patients were at higher risk of depression than male patients. Whether or not this is based on the regulation of gut microbiome to brain serotonin is worth more efforts and exploration.

## 5. Conclusion

The present study did not identify gender as an expected risk factor for GI dysfunctions in PD patients as expected. Investigating the reasons, we believe that, first of all, PD is a disease with multiple factors and complex mechanisms. Multiple systems may be involved, including the CNS, ENS, peripheral nervous system (PNS), and sleep-wake system. Each system may affect the other due to its existence. As a result, in addition to motor symptoms, the clinical manifestations are diverse, with a wide range of symptoms that are too complex to be clearly classified or to be independently distinguished. There does not seem to be one symptom or group of symptoms alone. Secondly, due to the relatively small sample size of this study, there may be some gap between the results and the real world. But, to some extent, we did find that female patients were more likely to develop gastroparesis. The close relationship between GI dysfunctions and PD deserves more in-depth discussion. On the other hand, the study unexpectedly found that gender is a risk factor for depression, which might provide guidance for clinical diagnosis and treatment and help to further explore the mechanisms of the disease.

In sum, study of gender differences in GI dysfunctions and other symptoms has potential to shed light on factors that contribute to the development of PD, to provide lens through which we may better view the mechanisms of the disease, and to point to new ways to reduce risk and develop treatment for both males and females suffering from PD.

### 5.1. Limitations

Our study has some limitations. First, the study was limited to the Chinese population and to one single-center. It has been reported that ethnicity is also one of the factors that possibly influence incidence, prevalence, and presentation in PD. One research indicated that NMSS scores were significantly higher in Asian (total score and domains “mood/apathy”) than White individuals [44]. Another research reported that the pattern of nonmotor symptoms among Chinese population is more marked in terms of cognition-based symptoms and activities of daily living [45]. Therefore, the results still require to be testified by expanded sample size with various races. Secondly, since data were collected through patients' recalls, recall bias might partially affect the accuracy of the results. Thirdly, diagnosis of some symptoms lacked objective detection or assessment methods.

## Figures and Tables

**Table 1:** General information of male and female PD patients.

	Total PD (*n* = 186)	M-PD (*n* = 95, 51.08%)	FM-PD (*n* = 91, 48.92%)	t/*Z*/*χ*^2^	*P*
Age (y)	67.53 ± 8.52	67.72 ± 9.55	67.37 ± 7.36	0.277	0.783
Education (y)	7.09 ± 4.49	8.07 ± 4.12	6.08 ± 4.65	3.105	0.002
Height (m)	1.64 ± 0.07	1.69 ± 0.06	1.58 ± 0.05	13.560	< 0.001
Weight (Kg)	63.30 ± 10.35	67.26 ± 10.40	59.17 ± 8.57	5.777	< 0.001
BMI	23.59 ± 3.22	23.59 ± 3.32	23.59 ± 3.13	−0.016	0.987
Occupational exposure to insecticidal/herbicides	7 (3.8%)	2 (2.1%)	5 (5.5%)	1.474	0.225
Hypertension	30 (16.1%)	14 (14.7%)	16 (17.6%)	0.278	0.598
Diabetes mellitus	17 (9.1%)	6 (6.3%)	11 (12.1%)	1.865	0.172
Smoke	25 (13.4%)	12 (12.6%)	13 (14.3%)	0.109	0.741
Alcohol drinking	19 (10.2%)	11 (11.6%)	8 (8.8%)	0.394	0.530
LED (mg)	400 (300–550)	375 (300–550)	437.5 (300–550)	−0.804	0.422
Disease duration (m)	48 (21.75–84)	48 (23–84)	48 (18–88)	−0.241	0.810

PD: Parkinson's Disease; M-PD: male patients with PD; FM-PD: female patients with PD; BMI: body mass index; LED: daily levodopa-equivalent dose.

**Table 2:** GI dysfunctions in M-PD and FM-PD group (%).

GI dysfunction	Total PD (*n* = 186)	M-PD group (*n* = 95)	FM-PD (*n* = 91)	*χ* ^2^	*P*
Mouth pain	16 (8.6%)	11 (11.58%)	5(5.50%)	2.1885	0.139
Dry mouth	110 (59.14%)	55 (57.89%)	55(60.44%)	0.1246	0.724
Drooling	24 (12.9%)	13 (13.68%)	11 (12.09%)	0.105	0.745
Dysgeusia	68 (36.56%)	34 (35.79%)	34 (37.36%)	0.0496	0.824
Dysphagia	38 (20.43%)	22 (23.16%)	16 (17.58%)	0.888	0.346
Gastroparesis					
Early satiety	44 (23.66%)	16 (16.84%)	28 (30.77%)	4.9922	0.025
Loss of appetite	64 (34.4%)	22 (23.16%)	42 (46.15%)	18.8909	0.001
Nausea	19 (10.21%)	6 (6.32%)	13 (14.29%)	3.219	0.073
Vomiting	8 (4.3%)	3 (3.16%)	5 (5.49%)	0.6165	0.432
Bowel dysfunction					
Constipation	102 (54.84%)	48 (50.52%)	54 (59.34%)	0.1694	0.681
Loose stool	8 (4.3%)	3 (3.16%)	5 (5.49%)	0.6165	0.432
Fecal incontinence	0(0%)	0 (0%)	0 (0%)	-	-

PD: Parkinson's Disease; *M*-PD: male patients with PD; FM-PD: female patients with PD.

**Table 3:** Motor symptoms and other nonmotor symptoms in M-PD and FM-PD group (%).

	Total PD (*n* = 186)	M-PD (*n* = 95, 51.08%)	FM-PD (*n* = 91, 48.92%)	t/*Z*/*χ*2	P
Age of motor symptoms onset	62.01 ± 9.56	62.33 ± 9.91	61.68 ± 9.22	0.459	0.647
Type of motor symptom onset (case %)					
Tremor	92 (49.46%)	49 (53.3%)	43 (46.7%)	0.348	0.555
Nontremor	94 (50.54%)	46 (48.9%)	48 (51.1%)		
Location of motor symptom onset (case %)					
Left	88 (47.31%)	43 (48.9%)	45 (51.1%)	4.247	0.120
Right	84 (45.16%)	48 (57.1%)	36 (42.9%)		
Dual side	14 (7.53%)	4 (28.6%)	10 (71.4%)		
Phenotype of motor symptom (case %)					
TD	52 (27.96%)	26 (50.0%)	26 (50.0%)	0.458	0.795
AR	47 (25.27%)	26 (55.3%)	21 (44.7%)		
Mixed	87 (46.77%)	43 (49.4%)	44 (50.6%)		
H-Y (onstage)	2.25 ± 1.05	2.20 ± 0.95	2.32 ± 1.15	−0.765	0.445
UPDRS-I	9 (4.5–15)	9 (4–15)	9.5 (5.25–19.0)	−0.821	0.413
UPDRS-II	14 (7.5–26.5)	13 (9–22.5)	16 (5.25–32.25)	−0.983	0.329
UPDRS-III	34 (18.5–56.5)	32 (17–51.5)	40.5 (21.25–65.75)	−2.131	0.034
UPDRS-IV	0 (0–1)	0 (0–0)	0 (0–3.5)	−1.718	0.09
PQSI (scores)	8 (3–13)	8 (4–11)	7 (3.25–10.0)	−1.651	0.10
ESS (scores)	5 (1–11.0)	6 (1.5–11.0)	9 (1.0–15.0)	−0.796	0.427
RLS	2.0 (0.0–5.0)	1.0 (0.0–5.0)	3.0 (0.0–6.0)	0.047	0.962
RBD	44 (23.6%)	17 (18.9%)	26 (28.6%)	2.0	0.157
HAMA (scores)	3.0 (1.0–8.0)	3.0 (0.0–8.0)	5.0 (0.0–15.0)	−1.918	0.057
HAMD (scores)	5.0 (1–13.0)	5.0 (1.5–10.0)	11.0 (1.0–22.0)	−2.373	0.033
MoCA (scores)	16.5 (11.0–22.0)	17.0 (9.0–20.0)	16.0 (12.0–24.0)	−1.459	0.149
MMSE (scores)	26.0 (20.0–28.0)	26.0 (21.0–28.0)	24.0 (20.0–28.0)	0.254	0.800

PD: Parkinson's Disease; M-PD: male patients with PD; F-PD: female patients with PD; TD: tremor-dominant subtype; AR: akinetic-rigid subtype; mixed: mixed subtype; H&Y: Hoehn-Yahr stage; UPDRS -I: United Parkinson's Disease Ranking Scale Part-I; UPDRS III: United Parkinson's Disease Ranking Scale part-III; PSQI: Pittsburgh Sleep Quality Index; ESS: Epworth Sleepiness Scale; RBD: Rapid Eye Movement (REM) Sleep Behavior Disorder; RLS: Restless Legs Syndrome; HAMA: Hamilton's Anxiety Scale; HAMD: Hamilton's Depression Scale; MMSE: Minimental State Examination; MoCA: Montreal Cognitive Assessment.

**Table 4:** Logistic Regression analysis of symptoms associated with gender in patients with PD.

Variables	Beta	Wald	*P*	OR	95% C.I
HAMD	−3.383	5.508	0.019	0.682	0.495–0.939

## Data Availability

The data are available from the corresponding author upon request.
